# Osteomalacia as a Complication of Intravenous Iron Infusion: A Systematic Review of Case Reports

**DOI:** 10.1002/jbmr.4558

**Published:** 2022-05-07

**Authors:** Tatiane Vilaca, Nalini Velmurugan, Christopher Smith, Bo Abrahamsen, Richard Eastell

**Affiliations:** ^1^ Department of Oncology and Metabolism University of Sheffield Sheffield UK; ^2^ Department of Medicine Holbæk Hospital Holbæk Denmark; ^3^ Open Patient Data Explorative Network, Department of Clinical Research University of Southern Denmark Odense Denmark; ^4^ Denmark and NDORMS University of Oxford Oxford UK

**Keywords:** IRON INFUSION, FGF‐23, HYPOPHOSPHATEMIA, OSTEOMALACIA, FRACTURES

## Abstract

Randomized control trials (RCTs) have shown that certain intravenous iron preparations can induce high levels of fibroblast growth factor 23 (FGF‐23) and persistent hypophosphatemia. Repeated iron infusions may lead to prolonged hypophosphatemia and osteomalacia events not captured by RCTs. Several previous case reports have described skeletal adverse effects after repeated iron infusions. To characterize these effects, we conducted a systematic review of case reports. MEDLINE, Embase, Web of Science, and Cochrane databases were searched in March 2021. We selected case reports of patients ≥16 years old. Study quality was assessed using the tool from Murad and colleagues. We report the results in a narrative summary. We identified 28 case reports, reporting 30 cases. Ages ranged from 28 to 80 years (median 50 years). Most patients (*n* = 18) received ferric carboxymaltose (FCM), whereas 8 received saccharated ferric oxide (SFO) and 3 received iron polymaltose (IPM). All but 2 cases had more than five infusions (range 2 to 198, median 17). The lowest phosphate levels ranged from 0.16 to 0.77 mmol/L (median 0.36 mmol/L). Intact FGF‐23 (iFGF‐23) was high when measured. Serum 25OH vitamin D was low in 10 of 21 cases measured and 1,25(OH)_2_ vitamin D in 12 of 18. Alkaline phosphatase was high in 18 of 22 cases. Bone or muscle pain was reported in 28 of the 30 cases. Twenty patients had pseudofractures, 9 had fractures, and 6 patients had both. All 15 available bone scans showed focal isotope uptake. Case reports tend to report severe cases, so potential reporting bias should be considered. Osteomalacia is a potential complication of repeated iron infusion, especially in patients with gastrointestinal disorders receiving prolonged therapy. Pain and fractures or pseudofractures are common clinical findings, associated with low phosphate, high iFGF‐23, high alkaline phosphatase, and abnormal isotope bone scan. Discontinuing or switching the iron formulation was an effective intervention in most cases. © 2022 The Authors. *Journal of Bone and Mineral Research* published by Wiley Periodicals LLC on behalf of American Society for Bone and Mineral Research (ASBMR).

## Introduction

Phosphate is one of the main components of the mineral bone compartment, and adequate serum levels are required for normal mineralization. Severe malnutrition and increased renal loss of phosphate are common causes of chronic phosphate depletion. Persistent hypophosphatemia leads to osteomalacia, a lack of mineralization of bone matrix.^(^ ^1^, ^2^ ^)^ Clinically, osteomalacia presents as muscle weakness and bone pain, biochemically as elevated alkaline phosphatase activity (ALP), and radiologically as Looser's zones (pseudofracture).^(^ ^2^ ^)^

Physiologically, fibroblast growth factor 23 (FGF‐23) is a key positive regulator of renal phosphate excretion in response to elevated phosphate levels. Inappropriately elevated intact FGF‐23 activity is associated with phosphate depletion and adverse bone and neuromuscular outcomes.^(^ ^2^ ^)^ Hypophosphatemic diseases, such as X‐linked hypophosphatemia and tumor‐induced osteomalacia, are characterized by high levels of FGF‐23. In X‐linked hypophosphatemia, FGF‐23 is not properly regulated because of a mutation on the PHEX gene, resulting in FGF‐23 overactivity. Conversely, in the rare tumor‐induced osteomalacia, FGF‐23—and occasionally other phosphatonins—is produced by mesenchymal phosphaturic tumors. In recent randomized controlled trials of up to 5 weeks duration, intravenous iron therapy with ferric carboxymaltose (FCM) was shown to increase the concentration of circulating intact FGF23 (iFGF‐23),^(^ ^3^, ^4^, ^5^ ^)^ but the long‐term effects of repeated iron infusions and prolonged hypophosphatemia have not been investigated. These abnormalities could adversely affect the skeleton, but the epidemiology and pathophysiology remain incompletely understood. Several previous case reports have reported adverse effects on the skeleton after repeated iron intravenous infusions. To characterize these adverse effects, we conducted a systematic review of case reports addressing the question: Is osteomalacia observed in adults receiving iron infusions for anemia treatment?

## Materials and Methods

### Search strategy and selection criteria

This review was conducted in line with the principles from the Cochrane Handbook and the Centre for Reviews Dissemination Handbook.^(^ ^6^ ^)^ This report followed the Preferred Reporting Items for Systematic Reviews and Meta‐Analyses (PRISMA).^(^ ^7^ ^)^ The protocol was registered in PROSPERO CRD42021243237.

MEDLINE, Embase, Web of Science, and Cochrane databases were searched on March 22, 2021 combining terms for iron infusion and outcomes such as “hypophosphatemia,” “osteomalacia,” “fractures,” and “pseudofractures” and other bone‐related features. We used relevant MeSH and free text terms with no search limits. Reference lists of key articles,^(^ ^1^, ^8^ ^)^ a list of references on hypophosphatemia associated with iron infusions collated by Pharmacosmos, and experts in the field were also consulted. The full search strategy can be found in Supplemental Material S1.

We included case reports or case report series of osteomalacia associated with hypophosphatemia in people older than 16 years who received any form of intravenous iron infusion. We excluded randomized controlled trials, conference abstracts, studies not written in English, Danish, Norwegian, Spanish, Portuguese, or French, studies that only reported abnormalities not related to the skeletal system, and studies where bone abnormalities were not associated with hypophosphatemia. We conducted a narrative synthesis, including tabulation of study characteristics, and a description of the available data. We grouped studies by clinical criteria (presence of pseudofractures/fractures) because these are the main clinical outcomes of osteomalacia.

We defined osteomalacia as musculoskeletal pain, fractures, and/or pseudofractures associated with low phosphate. Serum phosphate levels in mg/dL were converted in mmol/L; serum 25OH vitamin D levels on ng/mL were converted to nmol/L, and serum calcium levels in mg/dL were converted in mmol/L using standard formulas. Some articles reported “insufficiency fractures.” In patients with biochemical changes suggesting hypophosphatemic osteomalacia, such as bone pain and low phosphate, if there were signs of a fracture without displacement it was considered a pseudofracture. If there was a displacement of the two ends of the fracture, then we referred to it as a “fracture.”^(^ ^9^ ^)^

### Data analysis

We uploaded retrieved records into Endnote and removed duplicates. Two reviewers independently conducted the data extraction, the quality assessment, and the data checking using standardized and piloted forms (Supplemental Material S2 and S3). For each study, we extracted the information about the author, date, country, age, sex, clinical features of the condition that led to iron deficiency, details about iron infusion (iron formulation, dose, and frequency), phosphate levels, clinical, imaging, and laboratory features after iron infusion, details of hypophosphatemia management, and patient outcome. Disagreements at any step were resolved through discussion or involvement of a third reviewer.

There is no standard quality assessment tool to assess the quality of case report studies. We used a tool proposed by Murad and colleagues, which assesses eight items categorized in four domains: selection, ascertainment, causality, and reporting.^(^ ^10^ ^)^

## Results

### Study selection

**Fig. 1:**
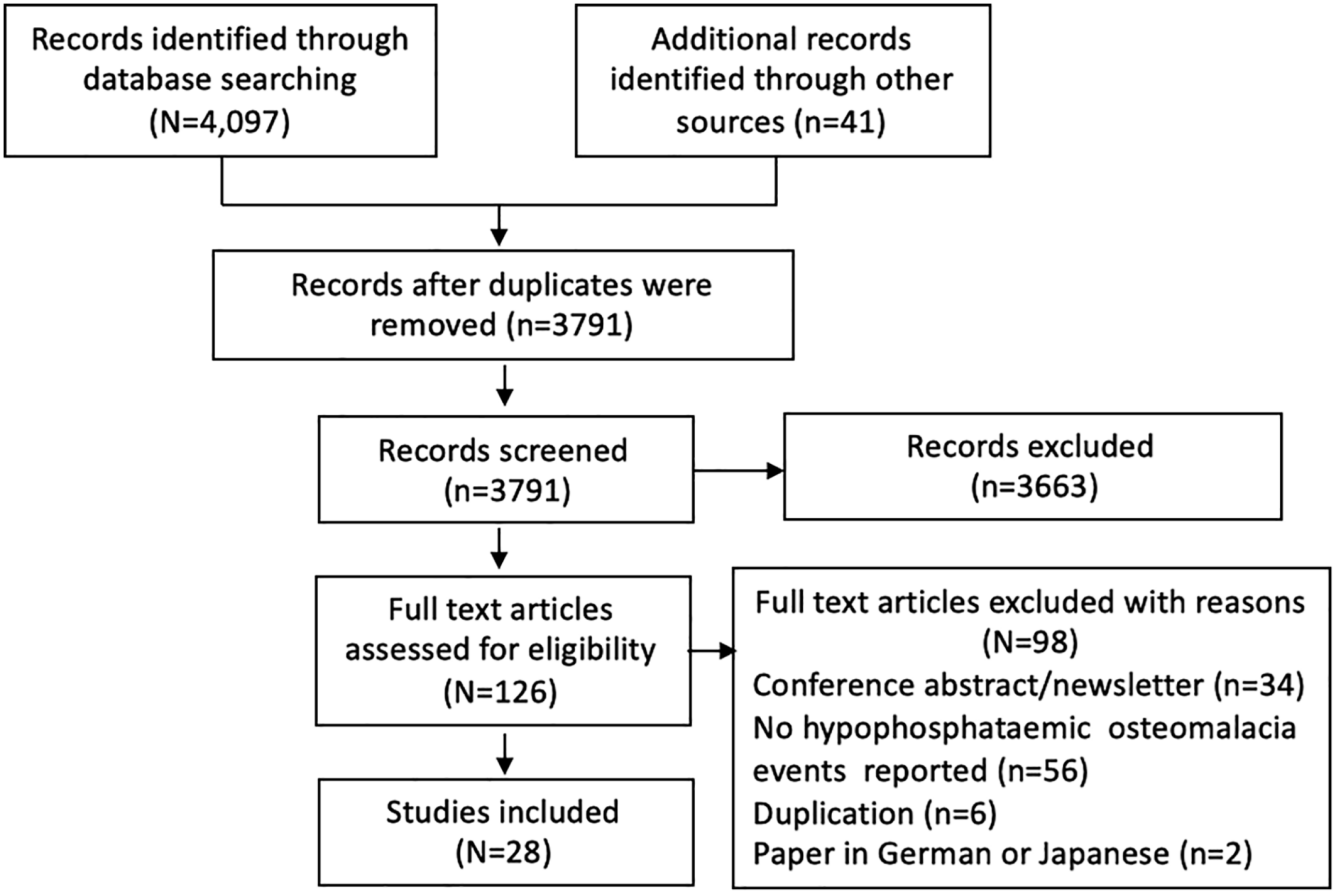
Study selection flow chart.

The study selection process is shown in Fig. 1. The initial selection process resulted in 4097 hits, and after duplicates were excluded, 3850 unique references were assessed, 128 underwent full‐text assessment, and 28 were included in this review.^(^ ^11^, ^12^, ^13^, ^14^, ^15^, ^16^, ^17^, ^18^, ^19^, ^20^, ^21^, ^22^, ^23^, ^24^, ^25^, ^26^, ^27^, ^28^, ^29^, ^30^, ^31^, ^32^, ^33^, ^34^, ^35^, ^36^, ^37^, ^38^ ^)^

### Case report characteristics

Twenty‐eight case reports were included,^(^ ^11^, ^12^, ^13^, ^14^, ^15^, ^16^, ^17^, ^18^, ^19^, ^20^, ^21^, ^22^, ^23^, ^24^, ^25^, ^26^, ^27^, ^28^, ^29^, ^30^, ^31^, ^32^, ^33^, ^34^, ^35^, ^36^, ^37^, ^38^ ^)^ reporting 30 cases. The vast majority (*n* = 26) were single case reports,^11^, ^12^, ^13^, ^15^, ^16^, ^17^, ^18^, ^19^, ^20^, ^21^, ^22^, ^23^, ^24^, ^25^, ^26^, ^27^, ^28^, ^29^, ^30^, ^32^, ^33^, ^34^, ^35^, ^36^, ^37^, ^38^ ^)^ and two manuscripts reported two cases.^(^ ^14^, ^31^ ^)^ The case reports were published between 1993 and 2020. Half of the case reports were from Europe (*n* = 14),^(^ ^11^, ^12^, ^13^, ^15^, ^17^, ^20^, ^21^, ^23^, ^25^, ^26^, ^28^, ^30^, ^32^, ^34^ ^)^ eight from Japan,^18^, ^22^, ^27^, ^29^, ^31^, ^36^, ^37^, ^38^ ^)^ and three from both Australia^(^ ^14^, ^16^, ^24^ ^)^ and the United States.^(^ ^19^, ^33^, ^35^ ^)^ The main characteristics of the patients in the case reports are shown in Table 1.

**Table 1:** Case Report Characteristics

Case report	Age (years)	G	Iron deficiency cause	IV iron	Cum dose (g)	s‐phos (mmol/L)	25OHD (nmol/L)	s‐ca (mmol/L)	FEP (%)	ALP (IU/L)^c^	Isotope bone scans
**Case‐reports reporting pseudo‐fractures and fractures**											
Amarnani 2020	32	M	Crohn's disease	FCM^f^	6.5	0.38	NR‐	NR	NR	218	NR^d^
Aubry‐Rozier 2017	38	M	Ulcerative colitis	FCM	NR	0.4	60	2.25	35	NR	Recent fractures in several ribs
Fang 2019	73	F	Gastric antral vascular ectasia	FCM	11.0	0.27	32	2.04	NR	229	Bilateral insufficiency fractures of the sacral wings
Klein 2017	57	M	Crohn's disease, short bowel	FCM^g^	19.5	0.77	25	2.12	NR	159	Multiple scattered foci along anterior and posterior aspects of the rib cage
Tozzi 2020	61	F	Hepatitis C, cirrhosis, varices	FCM	NR	0.58	77.0	2.22	NR	190	NR^d^
Yamamoto 2013	80	M	Short bowel	SFO	19.8	0.45	NR	2.02	NR	677	Abnormal accumulation in the bilateral knee and ankle joints and in multiple ribs
**Case reports reporting only pseudofractures**											
Bishay 2017	58	F	Multiple telangiectasia	IPM	17.0	0.43	54	2.4	24	125	Focal uptake at several ribs bilaterally (consistent with fractures), diffusely increased osteoblastic activity at the sternum, scapulae, long bones of the limbs, and costo‐chondral junctions
Callejas‐Moraga 2020	65	M	HHT	FCM	NR	0.39	54.5	2.32	6	356	Multiple hot spots located in several ribs, left scapula, bilateral sacral wings, ischiopubic and iliopubic rami, right femoral head, left tibia internal plateau, and both tarsi
Ishimaru 2017	77	F	Duodenum ulcer	SFO	8.3	0.55	NR	2.2	NR	507	NR^d^
Moore 2013	50	F	Iron loss in urine for unknown reason	FCM	NR	NR	Normal^a^	NR	NR	NR	Increased uptake in the anterior section of the frontal bone on both sides of the midline, in several ribs, both sacroiliac joints and proximally in the left tibia
Nomoto 2017	62	F	Crohn's disease, short bowel	SFO	NR	0.36	<7.5	2.15	NR	419	Increased uptake in the ribs, vertebrae, sacroiliac joints, knee joints, and ankle joints
Poursac 2015	57	F	HHT	FCM	NR	0.36	37.5	2.28	39	NR	NR^d^
Reyes 2017	45	M	Crohn's disease, short bowel	FCM	NR	0.21	75	2.04	NR	71	Acute fractures over multiple ribs (asymmetrical pattern), both pedicles of L_4_ vertebra, left sacral wing, femoral head, and metatarsals
Sangrós Sahún 2016	43	F	Menorrhagia secondary to uterine myomas	FCM	0.5	0.29	NR	NR	NR	189	A generalized increase in bone uptake compared with the background and multiple high‐intensity hyperactive foci in the 7th right costal margin, sacroiliac joints, knees, heads of the 2nd, 3rd, and 5th metatarsals of the right foot and cuneiform bone of the left foot, in addition to other focal uptake of lesser intensity in the left humeral diaphysis and both femurs
Schaefer 2017	45	M	Crohn's disease	FCM^h^	27.0	0.46	Normal^a^	NR	46	NR	NR^d^
Schouten 2009	38	M	Crohn's disease	IPM	20–40^e^	0.4	67	2.2	50	137	Multiple discrete areas of increased bony reaction suggesting fractures in multiple ribs, the sacrum, and feet
Segura 2014	67	M	Erosive gastritis	NR	NR	0.19	42.5	2.0	36	253	Hyper‐uptake in right internal tibial plateau, third metatarsal of right foot, dorsal spine, and D_3_ and L_2_ vertebrae, showing a process repair of the different fracture points, as well as foci of bilateral costo‐chondral hyperactivity compatible with new fractures
Suzuki 1993	58	F	NR	SFO	NR	0.45	NR	2.35	28	977	Abnormal uptake around the shoulders, ribs, hips, and ankles
Tournis 2018	31	M	Short bowel	FCM	NR	0.35	77.1	2.09	NR	72	NR
Yamamoto 2012	44	F	Uterine bleeding	SFO	NR	0.36	35	2.05	NR	206	Abnormal accumulation in the ribs and right femoral neck
**Case reports reporting only fractures**											
Bartko 2018	42	M	Crohn's disease, short bowel	FCM	1.8	0.5	87	1,27	17	180	NR
Bishay 2017	65	F	Gastric antral vascular ectasia	IPM	13.0	0.29	98	2.18	16	302	Increased focal uptake consistent with multiple rib fractures, increased metabolic activity involving the right distal radius, ribs, ankles, right inferior pubic ramus, and sacral wings
Urbina 2018	38	M	Crohn's disease	FCM	8.0	0.34	45	1.97	40	NR	NR
**Case reports that did not report fractures or pseudofractures** ^b^											
Fisher 2020	40	F	Celiac disease	FCM	2.0	0.23	NR	NR	NR	NR	NR
Lehmann 2018	NR	M	HHT	FCM	NR	0.32	89.6	2.24	NR	157	NR
Rodriguez 2019	45	F	Malabsorption after Roux‐en‐Y gastric bypass	FCM	NR	0.29	140	2.17	NR	NR	NR
Sato 1997	60	M	Hepatitis C, gi bleeding	SFO	> 25 g	0.16	29.7	2.0	NR	945	NR
Shimizu 2009 (2 cases)	43	F	Severe menorrhagia	SFO	11.0	0.32	NR	2.22	NR	565	NR
	52	F	Abnormal genital bleeding	SFO	2.0	0.45	NR	2.1	NR	830	NR
Vasquez‐Rios 2020	28	F	Uterine bleeding	FCM	1.5	0.32	NR	NR	21	NR	NR

G = gender; Cum iron dose = cumulative iron dose; s‐phos = serum phosphate; FEP = fractional P urinary excretion; M = male; F = female; NR = not reported; FCM = ferric carboxymaltose; SFO = saccharated ferric oxide; IPM = iron polymaltose; gi = gastrointestinal; HHT = hereditary hemorrhagic telangiectasia or Rendu‐Osler‐Weber syndrome.

Serum phosphate levels in mg/dL were converted in mmol/L; serum 25OH vitamin D levels on ng/mL were converted to nmol/L, and serum calcium levels in mg/dL were converted in mmol/L using standard formulas.

^a^ Vitamin D levels were reported to be normal, but the values were not reported.

^b^ There are not enough data to exclude the occurrence of either fracture or pseudofracture.

^c^ Several reference ranges reported (30–110; 30–120; 30–130; 35–110; 38–126; 39–117; 40–129; 40–150; 115–359).

^d^ Pseudofractures diagnosed by MRI.

^e^ Calculated based on dose and periodicity.

^f^ Followed by iron isomaltoside.

^g^ Followed by iron dextrose.

^h^ Followed by iron sucrose.

### Patient characteristics

Ages ranged from 28 to 80 years (median 50 years), with 6 patients younger than 40 years,^(^ ^11^, ^12^, ^29^, ^32^, ^34^, ^35^ ^)^ 20 patients between 40 and 70 years,^13^, ^14^, ^15^, ^17^, ^19^, ^21^, ^22^, ^23^, ^24^, ^25^, ^26^, ^27^, ^28^, ^30^, ^31^, ^33^, ^36^, ^38^ ^)^ and 3 older than 70 years old.^(^ ^16^, ^18^, ^37^ ^)^ In one case report, the age of the patient was not reported.^(^ ^20^ ^)^ Sixteen patients were females^(^ ^14^, ^16^, ^17^, ^18^, ^21^, ^22^, ^23^, ^25^, ^26^, ^33^, ^35^, ^36^, ^38^ ^)^ and 14 males.^(^ ^11^, ^12^, ^13^, ^15^, ^19^, ^20^, ^24^, ^27^, ^28^, ^29^, ^30^, ^32^, ^34^, ^37^ ^)^

Gastrointestinal diseases were by far the most common cause of iron deficiency (23 cases); 9 patients had inflammatory bowel disease (IBD),^(^ ^11^, ^12^, ^13^, ^19^, ^22^, ^24^, ^28^, ^29^, ^34^ ^)^ 5 had intestinal vascular ectasia,^(^ ^14^, ^15^, ^20^, ^23^ ^)^ 4 had short bowel (including one Roux‐en‐Y gastric bypass [RYGB]),^(^ ^22^, ^25^, ^32^, ^37^ ^)^ 3 patients had bleeding associated with cirrhosis,^(^ ^16^, ^27^, ^33^ ^)^ 1 had gastritis,^(^ ^30^ ^)^ 1 had a duodenal ulcer,^(^ ^18^ ^)^ and 1 patient had celiac disease.^(^ ^17^ ^)^ Five patients received intravenous iron for gynecological bleeding^(^ ^26^, ^31^, ^35^, ^36^ ^)^ and 2 for unknown reasons.^(^ ^21^, ^38^ ^)^

In 10 patients, no previous identifiable condition harmful to the skeleton was reported,^(^ ^14^, ^18^, ^20^, ^21^, ^26^, ^31^, ^33^, ^35^, ^38^ ^)^ whereas 9 patients had IBD,^(^ ^11^, ^12^, ^13^, ^19^, ^22^, ^24^, ^28^, ^29^, ^34^ ^)^ associated with malabsorption and/or glucocorticoid use, 4 patients had short bowel,^(^ ^22^, ^25^, ^32^, ^37^ ^)^ 3 had diabetes,^(^ ^14^, ^15^, ^30^ ^)^ and 1 had malabsorption associated with celiac disease.^(^ ^17^ ^)^ In 10 patients, vitamin D was reported to be low.^(^ ^12^, ^15^, ^16^, ^19^, ^22^, ^23^, ^27^, ^30^, ^34^, ^36^ ^)^ In these, 6 also had high parathyroid hormone (PTH),^(^ ^15^, ^16^, ^19^, ^27^, ^30^, ^36^ ^)^ 4 also had low calcium levels,^16^, ^22^, ^27^, ^34^ ^)^ and 6 also had high ALP,^(^ ^15^, ^16^, ^19^, ^22^, ^27^, ^30^ ^)^ suggesting vitamin D deficiency in 8 of them. In the remaining 2 patients, low vitamin D was associated with normal calcium.^(^ ^12^, ^23^ ^)^ In one of them, PTH was also normal, but ALP was not measured.^(^ ^23^ ^)^ In the remaining patient, neither PTH nor ALP were reported.^(^ ^12^ ^)^

Besides iron, the most common medication in use was anti‐TNF, used by 6 patients,^(^ ^12^, ^13^, ^22^, ^24^, ^28^, ^34^ ^)^ followed by mesalazine (used by 4),^13^, ^22^, ^24^, ^28^ ^)^ and current use of glucocorticoids (in 2 patients).^(^ ^24^, ^28^ ^)^ Two patients have previously received bisphosphonates^(^ ^15^, ^19^ ^)^ and one denosumab.^(^ ^16^ ^)^ Six patients were using calcium supplements^(^ ^13^, ^14^, ^15^, ^19^, ^25^, ^29^ ^)^ and 6 took cholecalciferol.^(^ ^13^, ^14^, ^15^, ^25^, ^28^ ^)^

### Infusions characteristics

The majority of patients received ferric carboximaltose (FCM) (*n* = 18),^11^, ^12^, ^13^, ^15^, ^16^, ^17^, ^19^, ^20^, ^21^, ^23^, ^24^, ^25^, ^26^, ^28^, ^32^, ^33^, ^34^, ^35^ ^)^ whereas 8 received saccharated ferric oxide (SFO),^(^ ^18^, ^22^, ^27^, ^31^, ^36^, ^37^, ^38^ ^)^ 3 received iron polymatose (IPM),^14^, ^29^ ^)^ and in 1 patient the iron therapy was not reported.^(^ ^30^ ^)^ All case reports related to SFO were from Japan. Two case reports related to IPM were from Australia, and the remaining one from Japan.^(^ ^18^, ^22^, ^27^, ^31^, ^36^, ^37^, ^38^ ^)^ Almost all patients recovered after discontinuing or switching iron therapy, but in Amarnani and colleagues, the patient remained hypophosphatemic after switching iron preparation and was treated with burosumab.^(^ ^11^ ^)^ When the number of infusions was available, it varied between 2^(^ ^17^, ^35^ ^)^ and approximately 198.^(^ ^31^ ^)^ However, SFO is a low‐dose iron preparation, leading to more frequent infusions, whereas FCM and IPM are high‐dose preparations. The number of doses including only FCM and IPM varied from 2 to 60, median of 17. The great majority of reported cases was in patients receiving at least five infusions, with only two case reports associated with only two infusions of FCM.^(^ ^17^, ^35^ ^)^ The interval between infusions also varied greatly from three times a week^(^ ^27^ ^)^ to once a year^(^ ^17^ ^)^ (median 1 month). The cumulative dose, when reported, varied from as little as 500 mg in 5 months^(^ ^26^ ^)^ to 40 grams^(^ ^29^ ^)^ (median 11 g), but 13 articles did not report this information.^(^ ^12^, ^15^, ^20^, ^21^, ^22^, ^23^, ^24^, ^25^, ^30^, ^32^, ^33^, ^36^, ^38^ ^)^

### Laboratory tests

The lowest phosphate level reported in each case ranged from 0.16^(^ ^27^ ^)^ to 0.77 mmol/L^(^ ^19^ ^)^ (median 0.36 mmol/L) or 0.35 to 2.39 mg/L (median 1.10 mg/L). Only 11 case reports reported enough data for some estimation of hypophosphatemia duration,^13^, ^17^, ^20^, ^23^, ^24^, ^25^, ^28^, ^29^, ^32^, ^33^, ^34^ ^)^ and it varied from 42 days^(^ ^17^ ^)^ to 5 years^(^ ^32^ ^)^ (median 36 months). In all the 15 cases where iFGF23 level was measured, it was high.^(^ ^11^, ^12^, ^14^, ^22^, ^23^, ^24^, ^28^, ^29^, ^31^, ^32^, ^33^, ^34^, ^36^, ^37^ ^)^ iFGF23 levels varied from 1.6^(^ ^31^ ^)^ to 7.8 times^(^ ^22^ ^)^ the upper limit of the reference range, median 3.75 times. Three articles reported high C‐terminal FGF‐23,^15^, ^19^, ^20^ ^)^ and one reported normal cFGF23 level.^(^ ^35^ ^)^ Twelve case reports reported fractional P urinary excretion,^12^, ^13^, ^14^, ^15^, ^23^, ^28^, ^29^, ^30^, ^34^, ^35^, ^38^ ^)^ and it was high in all of them, ranging from 5.9%^(^ ^15^ ^)^ to 50%^(^ ^29^ ^)^ (median 31%).

In 10 patients, vitamin D levels were reported to be low,^12^, ^15^, ^16^, ^19^, ^22^, ^23^, ^27^, ^30^, ^34^, ^36^ ^)^ whereas it was reported as normal in 11 patients^(^ ^13^, ^14^, ^20^, ^21^, ^24^, ^25^, ^28^, ^29^, ^32^, ^33^ ^)^ and not reported in 9^(^ ^11^, ^17^, ^18^, ^26^, ^31^, ^35^, ^37^, ^38^ ^)^). Eleven case reports reported low levels of 1,25OHD,^(^ ^13^, ^14^, ^20^, ^22^, ^24^, ^27^, ^29^, ^30^, ^31^, ^34^, ^37^ ^)^ whereas 6 reported normal levels.^(^ ^14^, ^21^, ^23^, ^31^, ^32^, ^36^ ^)^ In 1 patient taking calcitriol 0.25 μg twice a day, 1,25OHD was high, calcium was normal, vitamin D low, and PTH high.^(^ ^19^ ^)^ PTH was high in 13 case reports^(^ ^14^, ^15^, ^16^, ^19^, ^20^, ^24^, ^25^, ^26^, ^27^, ^30^, ^32^, ^36^, ^37^ ^)^ and normal in 13 case reports.^(^ ^13^, ^14^, ^18^, ^21^, ^22^, ^23^, ^28^, ^29^, ^31^, ^33^, ^34^, ^38^ ^)^ The remaining 4 cases did not report PTH levels.^(^ ^11^, ^12^, ^17^, ^35^ ^)^ In 10 case reports, serum calcium levels were low,^(^ ^16^, ^17^, ^20^, ^22^, ^24^, ^27^, ^28^, ^32^, ^34^, ^37^ ^)^ whereas in 16 it was normal^(^ ^12^, ^13^, ^14^, ^15^, ^18^, ^19^, ^21^, ^23^, ^25^, ^29^, ^30^, ^31^, ^33^, ^38^ ^)^ and 4 case reports did not report calcium levels.^(^ ^11^, ^26^, ^35^, ^36^ ^)^

In 18 case reports, ALP was high,^(^ ^13^, ^14^, ^15^, ^16^, ^18^, ^19^, ^20^, ^21^, ^22^, ^26^, ^27^, ^29^, ^30^, ^31^, ^33^, ^37^, ^38^ ^)^ in 4 it was normal,^(^ ^14^, ^24^, ^32^, ^36^ ^)^ and it was not reported in 8 cases.^(^ ^11^, ^12^, ^17^, ^23^, ^25^, ^28^, ^34^, ^35^ ^)^ ALP ranged from 71^(^ ^24^ ^)^ to 977 IU/L,^(^ ^38^ ^)^ median 229 IU/L. Bone ALP was high in 5 cases^(^ ^20^, ^22^, ^31^, ^37^ ^)^ and normal in 2.^(^ ^32^, ^36^ ^)^ Osteocalcin was measured in 2 cases^(^ ^13^, ^32^ ^)^ and was normal in both. PINP was also measured in 2 cases, and it was normal in 1^(^ ^32^ ^)^ and elevated in the other.^(^ ^14^ ^)^ CTX was also measured twice and was normal in both.^(^ ^14^, ^32^ ^)^ Only 1 case report reported TRACP‐5b level and it was high.^(^ ^22^ ^)^ Urine NTX was measured twice and was high in 1 case^(^ ^37^ ^)^ and normal in the other.^(^ ^36^ ^)^ Normal levels of urine pyridinoline and deoxypyridinoline were also reported once.^(^ ^27^ ^)^

### Osteomalacia signs and symptoms

Bone or muscle pain was reported in 28 of the 30 cases.^(^ ^11^, ^13^, ^14^, ^15^, ^16^, ^17^, ^18^, ^19^, ^20^, ^21^, ^23^, ^24^, ^25^, ^26^, ^27^, ^28^, ^29^, ^30^, ^31^, ^32^, ^33^, ^34^, ^35^, ^36^, ^37^, ^38^ ^)^ In 21 cases, the pain was associated with fractures or pseudofractures,^(^ ^11^, ^13^, ^14^, ^15^, ^16^, ^18^, ^19^, ^21^, ^23^, ^24^, ^26^, ^28^, ^29^, ^30^, ^32^, ^33^, ^34^, ^36^, ^37^, ^38^ ^)^ in 7 cases there was pain despite no fractures or pseudofractures reported,^17^, ^20^, ^25^, ^27^, ^31^, ^35^ ^)^ and in 2 cases pain was not reported despite pseudofractures.^(^ ^12^, ^22^ ^)^ The most common site for pain was the lower limbs (*n* = 19)^(^ ^11^, ^14^, ^15^, ^17^, ^18^, ^19^, ^20^, ^23^, ^24^, ^26^, ^27^, ^29^, ^31^, ^33^, ^34^, ^35^, ^37^, ^38^ ^)^ followed by the chest (*n* = 11),^(^ ^14^, ^15^, ^17^, ^19^, ^24^, ^26^, ^27^, ^29^, ^34^, ^36^, ^38^ ^)^ the pelvis^(^ ^15^, ^16^, ^21^, ^23^, ^26^, ^28^, ^34^, ^36^, ^38^ ^)^ and the back^(^ ^17^, ^21^, ^23^, ^24^, ^27^, ^29^, ^31^, ^34^, ^38^ ^)^ (*n* = 9 each), and finally the upper limbs^(^ ^14^, ^15^, ^17^, ^19^, ^26^ ^)^ or generalized pain^(^ ^13^, ^14^, ^25^, ^30^, ^32^ ^)^ in 5 case reports each.

Twenty patients had pseudofractures (14 of the chest,^(^ ^12^, ^14^, ^15^, ^19^, ^21^, ^22^, ^24^, ^26^, ^29^, ^30^, ^32^, ^36^, ^37^, ^38^ ^)^ 14 of the lower limbs,^(^ ^11^, ^15^, ^18^, ^21^, ^22^, ^24^, ^26^, ^28^, ^29^, ^30^, ^32^, ^33^, ^37^, ^38^ ^)^ 7 of the pelvis^(^ ^15^, ^16^, ^21^, ^22^, ^24^, ^26^, ^29^ ^)^, and 1 of the upper limb^(^ ^26^ ^)^). Nine patients had fractures (7 of the lower limbs,^(^ ^11^, ^13^, ^34^, ^36^ ^)^ 6 of the pelvis,^(^ ^11^, ^13^, ^14^, ^16^ ^)^ 4 of the ribs,^12^, ^14^, ^19^ ^)^ and 3 of the vertebra^(^ ^14^, ^16^, ^37^ ^)^). Six patients had both fractures and pseudofractures.^(^ ^11^, ^12^, ^16^, ^19^, ^33^, ^37^ ^)^ Muscular symptoms were present in 13 cases,^(^ ^12^, ^13^, ^14^, ^16^, ^17^, ^23^, ^25^, ^26^, ^27^, ^28^, ^31^, ^32^, ^35^ ^)^ gait disturbances in 6,^(^ ^13^, ^14^, ^16^, ^19^, ^23^, ^30^ ^)^ fatigue and malaise in 3,^(^ ^17^, ^24^, ^25^ ^)^ and hyperexcitability linked to hypocalcemia in 3.^(^ ^22^, ^26^, ^35^ ^)^

### Bone imaging

Bone mineral density was reported in 16 patients; in 5 it was normal,^(^ ^18^, ^20^, ^30^, ^36^, ^37^ ^)^ 3 had osteopenia,^14^, ^23^, ^24^ ^)^ and 8 had osteoporosis.^(^ ^13^, ^14^, ^15^, ^16^, ^19^, ^32^, ^33^, ^34^ ^)^ In 2 cases, bone mineral density (BMD) was also available after hypophosphatemia recovery and both showed increases; an 8% increase in femoral neck BMD and 19% increase in lumbar spine (LS) after 12 months was reported by Tournis^(^ ^32^ ^)^ and a substantial 63% increase in femoral neck (FN) BMD after 30 months was reported by Nomoto.^(^ ^22^ ^)^ An isotope bone scan was available in 15 cases, all abnormal. The most common areas affected were the ribs (in 12 patients),^(^ ^12^, ^14^, ^15^, ^19^, ^21^, ^22^, ^24^, ^29^, ^30^, ^36^, ^37^ ^)^ the pelvis (in 8 patients),^(^ ^15^, ^21^, ^22^, ^23^, ^24^, ^26^, ^29^, ^39^ ^)^ and the femur^(^ ^11^, ^15^, ^24^, ^26^, ^28^ ^)^ and the feet (in 5 patients each).^(^ ^15^, ^24^, ^26^, ^29^, ^30^ ^)^ Bone biopsy was performed in 4 cases^(^ ^13^, ^20^, ^24^, ^28^ ^)^ and showed osteomalacia in 3.^(^ ^13^, ^24^, ^28^ ^)^ Six patients underwent PET‐FDG^(^ ^15^, ^19^, ^20^, ^23^, ^24^, ^34^ ^)^ and 2 octreotide^(^ ^28^, ^34^ ^)^ scans looking for tumors as a source of FGF23 but all were negative.

### Treatment of osteomalacia

The most common treatments initiated were phosphate supplementation and active forms of vitamin D. Oral phosphate was prescribed in 18 cases^(^ ^11^, ^13^, ^14^, ^15^, ^16^, ^19^, ^20^, ^23^, ^24^, ^25^, ^26^, ^27^, ^28^, ^29^, ^30^, ^33^, ^34^, ^35^ ^)^ and intravenous phosphate in 4.^(^ ^11^, ^16^, ^17^, ^35^ ^)^ In 2 patients, milk was recommended as a source of phosphate.^(^ ^32^, ^38^ ^)^ Active vitamin D (oral alphacalcidol or calcitriol) was prescribed for 17 patients^(^ ^11^, ^13^, ^14^, ^16^, ^19^, ^20^, ^22^, ^23^, ^24^, ^27^, ^29^, ^30^, ^31^, ^32^, ^34^, ^35^ ^)^ and in 1 case intravenous calcitriol.^(^ ^35^ ^)^ However, there was little information available about the dose; Bishay and colleagues reported the use of 0.25 mcg/d of oral calcitriol,^(14^ ^)^ whereas Reyes and colleagues reported the use of calcitriol 0.25 mcg t.i.d.^(^ ^24^ ^)^ Vasquez‐Rios reported the use of 1 to 3 mcg/d calcitriol intravenously.^(^ ^35^ ^)^ Alphacalcidol dose was reported in 2 case reports and varied from 1 to 2 mcg/d.^(^ ^27^, ^32^ ^)^ Five patients received cholecalciferol^(^ ^14^, ^15^, ^26^, ^27^, ^29^ ^)^ and 5 received calcium supplementation.^(^ ^14^, ^15^, ^20^, ^26^, ^32^ ^)^ Three patients did not receive any treatment,^(^ ^12^, ^36^, ^37^ ^)^ whereas one with a non‐healing fracture was treated with teriparatide^(^ ^18^ ^)^ and another one with persistent symptomatic hypophosphatemia despite the change in iron intravenous formulation and intravenous phosphate infusion was treated with burosumab.^(^ ^11^ ^)^ All patients recovered from hypophosphatemia and osteomalacia, but the time to recovery varied substantially. For hypophosphatemia, recovery was observed from 2 weeks^(^ ^31^, ^35^ ^)^ to several months^(^ ^29^ ^)^ and for symptoms between 1^(^ ^37^ ^)^ and 6 months.^(^ ^18^ ^)^ Fracture healing was reported in 9 to 12 months in 4 case reports^(^ ^13^, ^18^, ^21^, ^36^ ^)^ and bone scan improvement in 6^(^ ^19^ ^)^ to 12^(^ ^26^ ^)^ months.

### Quality assessment

To assess the quality of the case reports, we used a tool proposed by Murad and colleagues, which assesses eight items categorized in four domains: selection, ascertainment, causality, and reporting.^(^ ^10^ ^)^ Results are reported in Table 2.For the selection, the tool asks if the patient represents the whole experience of the center on the disease. None of the case reports included in this review reported that this was their whole experience on bone adverse outcomes associated with hypophosphatemia. In regard to the ascertainment of the exposure and the outcome, all the case reports were based on clinical records, which is the highest possible quality of ascertainment.^(^ ^11^, ^12^, ^13^, ^14^, ^15^, ^16^, ^17^, ^18^, ^19^, ^20^, ^21^, ^22^, ^23^, ^24^, ^25^, ^26^, ^27^, ^28^, ^29^, ^30^, ^31^, ^32^, ^33^, ^34^, ^35^, ^36^, ^37^, ^38^ ^)^ Alternative causes that could explain the observation were clearly ruled out in 22 of the case reports.^(^ ^11^, ^13^, ^14^, ^15^, ^18^, ^19^, ^20^, ^22^, ^23^, ^24^, ^25^, ^26^, ^27^, ^28^, ^29^, ^30^, ^31^, ^32^, ^34^, ^36^ ^)^ Only 5 case reports reported a re‐challenge with the same iron infusion, resulting in hypophosphatemia again.^(^ ^13^, ^17^, ^29^, ^32^, ^34^ ^)^ Because the bone adverse events seem to be associated with chronic hypophosphatemia, we considered the worsening of signs and symptoms with cumulative dose as a dose–response effect. This was reported in 21 of the 30 case reports.^(^ ^11^, ^13^, ^14^, ^16^, ^18^, ^19^, ^20^, ^23^, ^24^, ^26^, ^27^, ^28^, ^29^, ^30^, ^31^, ^32^, ^34^, ^36^, ^38^ ^)^ Because we only included case reports that reported signs or symptoms of osteomalacia associated with hypophosphatemia, the follow‐up was long enough in all cases.^(^ ^11^, ^12^, ^13^, ^14^, ^15^, ^16^, ^17^, ^18^, ^19^, ^20^, ^21^, ^22^, ^23^, ^24^, ^25^, ^26^, ^27^, ^28^, ^29^, ^30^, ^31^, ^32^, ^33^, ^34^, ^35^, ^36^, ^37^, ^38^ ^)^ Finally, we considered that enough details were reported in 21 of the 30 cases.^(^ ^12^, ^13^, ^14^, ^15^, ^16^, ^18^, ^19^, ^20^, ^22^, ^23^, ^24^, ^26^, ^27^, ^28^, ^29^, ^32^, ^34^, ^36^, ^37^, ^38^ ^)^

**Table 2:** Quality Assessment Following Murad Et Al.

Case report	1. Does the patient(s) represent(s) the whole experience of the center?	2. Was the exposure adequately ascertained?	3. Was the outcome adequately ascertained?	4. Were other alternative causes that may explain the observation ruled out?	5. Was there a challenge/re‐challenge phenomenon?	6. Was there a dose–response effect?	7. Was follow‐up long enough for outcomes to occur?	8. Is the case(s) described with sufficient details?
Amarnani 2020	No	Yes	Yes	Yes	No	Yes	Yes	No
Aubry‐Rozier 2017	No	Yes	Yes	No	No	No	Yes	Yes
Bartko 2018	No	Yes	Yes	Yes	Yes	Yes	Yes	Yes
Bishay 2017^a^	No	Yes	Yes	Yes	No	Yes	Yes	Yes
	No	Yes	Yes	Yes	No	Yes	Yes	Yes
Callejas‐Moraga 2020	No	Yes	Yes	Yes	No	No	Yes	Yes
Fang 2019	No	Yes	Yes	No	No	Yes	Yes	Yes
Fisher 2020	No	Yes	Yes	No	Yes	No	Yes	No
Ishimaru 2017	No	Yes	Yes	Yes	No	Yes	Yes	Yes
Klein 2017	No	Yes	Yes	Yes	No	Yes	Yes	Yes
Lehmann 2018	No	Yes	Yes	Yes	No	Yes	Yes	Yes
Moore 2013	No	Yes	Yes	No	No	No	Yes	No
Nomoto 2017	No	Yes	Yes	Yes	No	No	Yes	Yes
Poursac 2015	No	Yes	Yes	Yes	No	Yes	Yes	Yes
Reyes 2017	No	Yes	Yes	Yes	No	Yes	Yes	Yes
Rodriguez 2019	No	Yes	Yes	Yes	No	No	Yes	No
Sangrós Sahún 2016	No	Yes	Yes	Yes	No	Yes	Yes	Yes
Sato 1997	No	Yes	Yes	Yes	No	Yes	Yes	Yes
Schaefer 2017	No	Yes	Yes	Yes	No	Yes	Yes	Yes
Schouten 2009	No	Yes	Yes	Yes	No	Yes	Yes	Yes
Segura 2014	No	Yes	Yes	Yes	No	Yes	Yes	No
Shimizu 2009^a^	No	Yes	Yes	Yes	No	Yes	Yes	No
	No	Yes	Yes	Yes	No	Yes	Yes	No
Suzuki 1993	No	Yes	Yes	No	No	Yes	Yes	Yes
Tournis 2018	No	Yes	Yes	Yes	Yes	Yes	Yes	Yes
Tozzi 2020	No	Yes	Yes	No	No	No	Yes	No
Urbina 2018	No	Yes	Yes	Yes	Yes	Yes	Yes	Yes
Vasquez‐Rios 2020	No	Yes	Yes	No	No	No	Yes	No
Yamamoto 1 2012	No	Yes	Yes	Yes	No	Yes	Yes	Yes
Yamamoto 2 2013	No	Yes	Yes	No	No	No	Yes	Yes

^a^ Two cases reported.

Question 1: Selection method unclear.

Questions 2 and 3: All case reports were based on clinical records.

Question 6: Worsening of the symptoms with cumulative dose considered dose–response effect.

## Discussion

This is the first systematic review of case reports of osteomalacia as a complication of intravenous iron infusion. We found 30 cases of ostemalacia associated with repeated iron infusions. In the vast majority of the patients, gastrointestinal diseases were the cause of iron deficiency, and in a few cases, iron deficiency was caused by gynecological bleeding. We observed osteomalacia in patients who received certain iron formulations (FCM, SFO, IPM) that have previously been linked to hypophosphatemia in randomized controlled trials and observational studies.^(^ ^1^, ^40^ ^)^ The mechanism seems to be an increase in FGF‐23.^(^ ^3^ ^)^ FGF‐23 leads to renal phosphate loss and decreased activation of vitamin D. The clinical picture was bone pain, fractures, and pseudofractures.^(^ ^2^, ^41^ ^)^ Most of the cases had an increase in ALP, and all isotope bone scans reported were abnormal. Treatment with phosphate and active forms of vitamin D seems to be of limited benefit, while discontinuing or switching iron preparation was the most effective intervention.

Abnormalities in FGF‐23 metabolism mediate hypophosphatemia associated with repeated iron infusions.^(^ ^3^, ^5^, ^41^, ^42^ ^)^ Experimental data suggest that iron deficiency increases FGF‐23 expression through action on hypoxia‐inducible factors (HIFs), HIF1a and HIF1b.^(^ ^43^, ^44^ ^)^ The increase in the production of intact FGF‐23 (iFGF‐23) is usually followed by an increase in the cleavage and generation of c‐FGF23 and N‐terminal fragments and has no impact on phosphate levels. However, some iron preparations (FCM, SFO, IPM) seem to decrease the physiological cleavage of iFGF‐23, resulting in high levels of iFGF‐23 and hypophosphatemia.^(^ ^45^ ^)^ The resulting hypophosphatemia might last for weeks to months.^(^ ^3^, ^5^, ^46^ ^)^ Some patients with severe iron deficiency require repeated infusions, which could lead to prolonged hypophosphatemia. In this review, most patients with osteomalacia had more than five infusions, suggesting that persistent hypophosphatemia is likely required for the development of osteomalacia.

Hypophosphatemia (mostly moderate or severe) was associated with other abnormalities in the phosphate homeostasis axis. The cut‐offs of 0.8, 0.6, and 0.3 mmol/L categorize mild, moderate, or severe hypophosphatemia.^(^ ^47^ ^)^ In our case series, the lowest phosphate levels reported were between 0.16 and 0.77 mmol/L, and 1 patient had mild, 20 had moderate, and 8 had severe hypophosphatemia (1 was not reported). RCTs that investigated the effects of FCM in phosphate metabolism have reported an increase in iFGF‐23, renal phosphate wasting, and PTH and a decrease in 1,25OHD and calcium.^(^ ^5^ ^)^ An observational study reported similar findings after IPM, except for the decrease in calcium levels.^(^ ^40^ ^)^ Case reports do not describe data systematically, but an increase in iFGF‐23 and phosphate wasting was found when measured. In addition, in 11 of 17 cases, 1,25OHD was decreased, in half of the cases when PTH was measured it was high, and serum calcium levels were low in 10 of 26 cases. Noteworthily, in the case reports, we were not able to capture variations in the blood tests within the normal range. For example, it is possible that PTH has increased in some patients, without becoming high. Therefore, similar findings were reported in RCTs, observational studies, and this review of case reports. The consistency of the findings suggests a strong association between the iron infusions and the phosphate homeostasis abnormalities and the resulting osteomalacia.

Intravenous iron is indicated for the treatment of iron deficiency (ID) and/or iron deficiency anemia (IDA). ID/IDA is commonly caused by gastrointestinal (GI) blood loss, inflammatory bowel disease (IBD), heavy uterine bleeding, and postpartum hemorrhage.^(^ ^47^ ^)^ A systematic review and meta‐analysis of prospective studies with FCM and ferric derisomaltose (FDI) suggested that the risk factors for hypophosphatemia are the type of iron preparation (higher with FCM), the degree of iron deficiency (more likely if ferritin or transferrin saturation are low), and kidney function (more likely if renal function is normal).^(^ ^47^ ^)^

FCM, SFO, and IPM were prescribed in 18, 8, and 3 cases (62%, 28%, and 10%), respectively, three forms of iron preparation previously associated with hypophosphatemia.^(^ ^1^ ^)^ Another systematic review reported increased rates of hypophosphatemia for FCM and iron sucrose but not iron dextran or ferumoxytol.^(^ ^48^ ^)^ The rising concerns in regard to osteomalacia associated with intravenous iron preparations have led to the inclusion of a warning on hypophosphatemic osteomalacia in FCM product information and a safety update by the Medicines and Healthcare Products Regulatory Agency (MHRA) on the risk of osteomalacia after FCM in the United Kingdom.^(^ ^49^ ^)^

In this review, there were no cases of osteomalacia associated with chronic kidney disease (CKD). Because CKD prevents hypophosphatemia, the increase in FGF‐23 associated with iron infusion is not followed by hypophosphatemia and osteomalacia associated with hypophosphatemia after repeated iron infusion is not observed.

Patients with comorbidities that could have detrimental effects on bone homeostasis such as malabsorption, corticosteroid use, and vitamin D deficiency might be at higher risk of osteomalacia. In this review, osteomalacia was associated with gastrointestinal disease in 23 of 30 cases. Low vitamin D status was reported in 10 of 21 cases (48%). The definition of low was left to the author; if we had used the threshold by the Institute of Medicine of 50 nmol/L, then 8 of 19 would have been low.^(^ ^50^ ^)^ This might have contributed to the low phosphate through secondary hyperparathyroidism. Several of the patients with low vitamin D had other biochemical features of vitamin D deficiency, such as low serum calcium and high ALP and PTH. ALP is usually high in osteomalacia, but osteomalacia with normal ALP have been previously reported in patients with malabsorption, often associated with low calcium.^(^ ^51^ ^)^ ALP was high in 18 of the 22 cases in which it was reported (82%). In the 4 cases where ALP was normal, 2 had abnormal bone scans^(^ ^24^, ^36^ ^)^ and the other 2 had rib pseudofractures.^(^ ^14^, ^32^ ^)^ Therefore, osteomalacia might be present with normal ALP. Two of these cases had malabsorption and low calcium.^(^ ^24^, ^32^ ^)^ We found IBD associated with osteomalacia in 30% of cases; IBD is likely to be associated with malabsorption of calcium and phosphate and vitamin D deficiency, all of which would likely increase the risk of osteomalacia. IBD is often treated with glucocorticoids, and 2 patients were in use of corticosteroids when osteomalacia was reported, which may have contributed to bone fragility, if not osteomalacia.

Osteomalacia presented as pain, fractures, and pseudofractures. Pseudofractures (or Looser's zones) are the radiological hallmark of osteomalacia, and these can go onto complete fracture. As expected, a large proportion of patients had either pseudofractures or fractures, and these were the likely sources of the bone pain. Pseudofractures are best identified by the isotope bone scan. This scan was performed in 15 cases and all of these showed focal increased uptake that can be due to pseudofractures or fractures. There were 7 patients who had pain but no fracture or pseudofractures. However, in every case, there was no imaging to test for fractures, so non‐diagnosed fractures cannot be ruled out.

Some patients were reported to have osteopenia (*n* = 3) and osteoporosis (*n* = 8). Although bone mineral density was reported to be low, it is important to highlight that osteomalacia could have contributed to these findings, due to the unmineralized osteoid matrix. This is confirmed by the increase in BMD observed after the recovery from hypophosphatemia reported in 2 cases.

Several strategies were used to treat hypophosphatemia and its consequences. Oral or intravenous phosphate and active metabolites of vitamin D (calcitriol or alfacalcidol) were often prescribed, but they were not able to normalize serum phosphate. Care needs to be taken with the active metabolites of vitamin D as they can cause hypercalcemia and hypercalciuria with nephrocalcinosis and nephrolithiasis. The latter could be of clinical importance in patients with IBD as they may have enteric hyperoxaluria and so be prone to calcium oxalate kidney stones. Vitamin D deficiency should be corrected by the administration of vitamin D3.

The most efficient intervention for recovery was discontinuing the intravenous iron infusions. That was not always possible, and sometimes the iron preparation was switched. This was followed by improvement in hypophosphatemia in most cases. In one case, hypophosphatemia persisted and osteomalacia progressed despite switching the iron preparation. That was a patient with severe Crohn's disease who received FCM, FDI, and then burosumab. There is not much data on the sequential use of iron preparations. In an observational study, 32 patients received FCM and FDI. FCM caused a greater reduction in serum phosphate than FDI and the median of phosphate levels returned to baseline after 5 weeks with FDI and 10 weeks after FCM.^(^ ^52^ ^)^ We do need further research on the effect of switching between iron preparations on serum phosphate. Burosumab is an antibody to FGF‐23 and when it was used, the symptoms improved along with serum phosphate. However, this treatment is not licensed for this indication. Finally, bisphosphonates are not indicated for osteomalacia.^(^ ^1^ ^)^

**Fig. 2:**
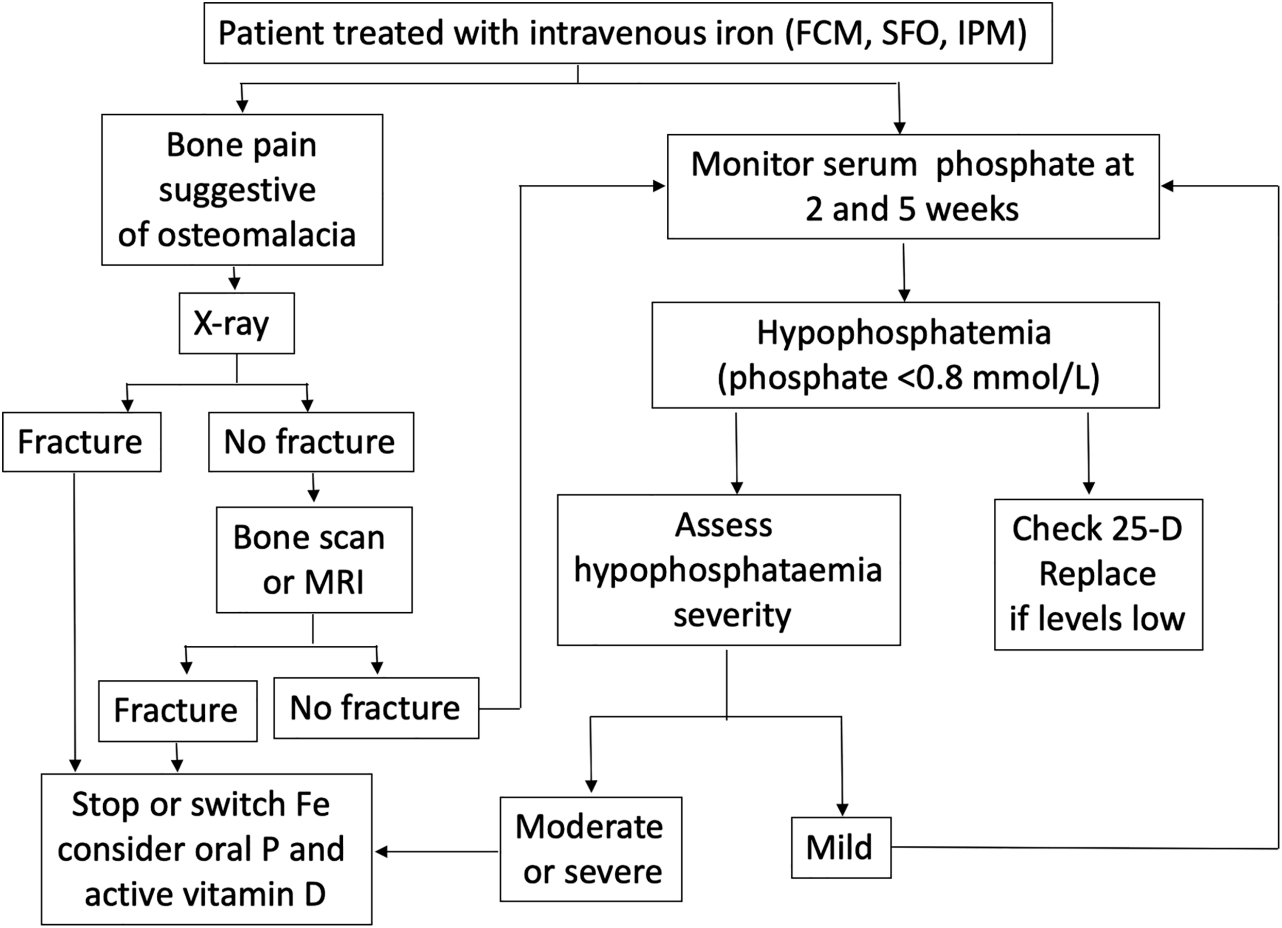
Proposed algorithm for diagnosis and management of osteomalacia associated with repeated iron infusions.

MHRA UK recommends to monitor serum phosphate levels in patients treated with multiple high‐dose administrations or those on long‐term treatment and in those with preexisting risk factors for hypophosphatemia, and reevaluate FCM treatment in patients with persistent hypophosphatemia.^(^ ^49^ ^)^ Based on these recommendations and the findings of this review, we would propose the management algorithm shown in Fig. 2 for patients receiving repeated iron infusions. Our recommendation is to check phosphate levels at 2 and 5 weeks after infusion (based on data from the RCTs) and perform skeletal imaging if bone pain develops, also after the 5‐week period. In those cases, also recheck phosphate levels.

Recently, a review has discussed the iron‐phosphate axis and the complications of intravenous iron‐induced hypophosphatemia, including osteomalacia.^(^ ^41^ ^)^ Differences in the results between the two reviews are probably associated with differences in the methodology applied; the review by Schaefer and colleagues is a narrative review;^(41^ ^)^ this is a PROSPERO registered and prespecified systematic review, which included only peer‐reviewed manuscripts.

This study has limitations. Case reports are reported according to local practice. Therefore, different teams might assess the patients in different ways, using different tests and management. There is no standardization of how the case is investigated, conducted, or reported. Because there is no systematic approach, in the absence of some information, it is impossible to know if this was a negative finding not reported or if it was not investigated. For example, in the many cases that did not report fractures, it is impossible to know if there were no fractures or if fractures were not investigated. In addition, case reports tend to describe severe cases and mild forms are less likely to be reported, which might lead to a publication bias. Case reports are considered low‐grade evidence. Because this is a systematic review of case reports, caution should be taken while interpreting the results. However, case reports are the first line of information and can suggest associations. Moreover, consistent findings in multiple case reports might suggest a pattern and help to understand the potential underlying mechanisms. Finally, several case reports might help to draw a bigger picture and to plan further research steps using appropriate methodology.

We conducted a systematic review of case reports of osteomalacia after repeated iron infusion. We found 30 cases of osteomalacia characterized by musculoskeletal pain, fractures, and pseudofractures. Not all cases presented high alkaline phosphatase, but iFGF‐23 and isotope bone scans were abnormal whenever reported. Osteomalacia was associated with FCM, SFO, and IPM, iron formulations that have been shown to increase iFGF‐23 and lead to significant hypophosphatemia. Phosphate supplementation and active forms of vitamin D were used for treatment, but the most effective intervention was discontinuing or switching intravenous iron preparation. In patients receiving repeated iron infusions, musculoskeletal pain can be a symptom of osteomalacia and it should be investigated.

## Disclosures

BA has received institutional research grants from UCB, Kyowa‐Kirin, Pharmacosmos, and Novartis and consulting or speaker fees from UCB, Kyowa‐Kirin, Pharmacosmos, and Amgen. RE has received consultancy funding from IDS, Sandoz, Nittobo, Samsung, Haoma Medica, CL Bio, Biocon, Amgen, Hindustan Unilever, Pharmacosmos, Takeda, and Viking and grant funding from Nittobo, Roche, Pharmacosmos, and Alexion. All other authors state that they have no conflicts of interest.

## AUTHOR CONTRIBUTIONS

**Tatiane Vilaca:** Conceptualization; data curation; formal analysis; methodology; writing – original draft; writing – review and editing. **Nalini Velmurugan:** Data curation; formal analysis; methodology; writing – review and editing. **Christopher Smith:** Data curation; methodology; writing – review and editing. **Bo Abrahamsen:** Conceptualization; formal analysis; funding acquisition; writing – review and editing. **Richard Eastell:** Conceptualization; formal analysis; funding acquisition; writing – review and editing.

### PEER REVIEW

The peer review history for this article is available at https://publons.com/publon/10.1002/jbmr.4558.

## Supporting information


**Appendix S1:** Supporting Information

**Appendix S2:** Supporting Information

**Appendix S3:** Supporting Information

## Data Availability

Data derived from public domain resources

## References

[1] Zoller H , Schaefer B , Glodny B . Iron‐induced hypophosphatemia: an emerging complication. Curr Opin Nephrol Hypertens. 2017;26(4):266‐275. 28399017 10.1097/MNH.0000000000000329

[2] Fukumoto S . FGF23‐related hypophosphatemic rickets/osteomalacia: diagnosis and new treatment. J Mol Endocrinol. 2021;66(2):R57–65. 33295878 10.1530/JME-20-0089

[3] Wolf M , Rubin J , Achebe M , et al. Effects of iron isomaltoside vs ferric carboxymaltose on hypophosphatemia in iron‐deficiency anemia: two randomized clinical trials. JAMA. 2020;323(5):432‐443. 32016310 10.1001/jama.2019.22450 PMC7042864

[4] Wolf M , Koch TA , Bregman DB . Effects of iron deficiency anemia and its treatment on fibroblast growth factor 23 and phosphate homeostasis in women. J Bone Miner Res. 2013;28(8):1793‐1803. 23505057 10.1002/jbmr.1923

[5] Wolf M , Chertow GM , Macdougall IC , Kaper R , Krop J , Strauss W . Randomized trial of intravenous iron‐induced hypophosphatemia. JCI Insight. 2018;3(23):1‐12. 10.1172/jci.insight.124486 PMC6328019 30518682

[6] Higgins JPT , Green S , (editors). Cochrane Handbook for Systematic Reviews of Interventions Version 5.1.0 [updated March 2011]. The Cochrane Collaboration, 2011. Available from www.handbook.cochrane.org

[7] Moher D , Liberati A , Tetzlaff J , Altman DG . Preferred reporting items for systematic reviews and meta‐analyses: the PRISMA statement. PLoS Med. 2009;6(7):e1000097. 19621072 10.1371/journal.pmed.1000097 PMC2707599

[8] Schaefer B , Tobiasch M , Viveiros A , et al. Hypophosphatemia after treatment of iron deficiency with intravenous ferric carboxymaltose or iron isomaltoside—a systematic review and meta‐analysis. Br J Clin Pharmacol. 2021;87(5):2256‐2273. 33188534 10.1111/bcp.14643 PMC8247006

[9] Haffner D , Emma F , Eastwood DM , et al. Clinical practice recommendations for the diagnosis and management of X‐linked hypophosphatemia. Nat Rev Nephrol. 2019;15(7):435‐455. 31068690 10.1038/s41581-019-0152-5 PMC7136170

[10] Murad MH , Sultan S , Haffar S , Bazerbachi F . Methodological quality and synthesis of case series and case reports. BMJ Evid Based Med. 2018;23(2):60‐63. 10.1136/bmjebm-2017-110853 PMC6234235 29420178

[11] Amarnani R , Travis S , Javaid MK . Novel use of burosumab in refractory iron‐induced FGF23‐mediated hypophosphataemic osteomalacia. Rheumatology (Oxford). 2020;59(8):2166‐2168. 31930323 10.1093/rheumatology/kez627 PMC7382597

[12] Aubry‐Rozier B , Stoll D , Gonzalez‐Rodriguez E . Phosphocalcic anomalies and bone fragility keys for the general practitioner. Rev Med Suisse. 2017;13(559):838‐843. 28727340

[13] Bartko J , Roschger P , Zandieh S , Brehm A , Zwerina J , Klaushofer K . Hypophosphatemia, severe bone pain, gait disturbance, and fatigue fractures after iron substitution in inflammatory bowel disease: a case report. J Bone Miner Res. 2018;33(3):534‐539. 29068481 10.1002/jbmr.3319

[14] Bishay RH , Ganda K , Seibel MJ . Long‐term iron polymaltose infusions associated with hypophosphataemic osteomalacia: a report of two cases and review of the literature. Ther Adv Endocrinol Metab. 2017;8(1–2):14‐19. 28203361 10.1177/2042018816678363 PMC5298444

[15] Callejas‐Moraga EL , Casado E , Gomez‐Nunez M , Caresia‐Aroztegui AP . Severe osteomalacia with multiple insufficiency fractures secondary to intravenous iron therapy in a patient with Rendu‐Osler‐weber syndrome. Bone Rep. 2020;13:1‐5. 10.1016/j.bonr.2020.100712 PMC7475229 32923530

[16] Fang W , Bloom S , Garg M , McMahon LP . Symptomatic severe hypophosphatemia after intravenous ferric carboxymaltose. JGH Open. 2019;3(5):438‐440. 31633052 10.1002/jgh3.12150 PMC6788463

[17] Fisher S , Jonker L . Ferric carboxymaltose (Ferinject) associated hypophosphatemia: case report illustrating the need for increased awareness to minimise incidence and risk. Acute Med. 2020;19(2):102‐105. 32840261

[18] Ishimaru D , Sumi H . A case of an insufficiency fracture of the medial proximal tibia secondary to osteomalacia associated with long‐term saccharated ferric oxide administration. Case Rep Orthop. 2017;2017:1675654. 28744387 10.1155/2017/1675654 PMC5514337

[19] Klein K , Asaad S , Econs M , Rubin JE . Severe FGF23‐based hypophosphataemic osteomalacia due to ferric carboxymaltose administration. BMJ Case Rep. 2018;2018:1‐5. 10.1136/bcr-2017-222851 PMC5775795 29298794

[20] Lehmann G , Wolf G Hypophosphatämie und FGF‐23‐Erhöhung bei normaler Nierenfunktion. Hypophosphataemia and FGF‐23‐Increase with Normal Renal Function An unusual constellation. The Nephrologist. 2018;13:186‐188.

[21] Moore KLF , Kildahl‐Andersen O , Kildahl‐Andersen R , Tjonnfjord GE . Uncommon adverse effect of a common medication. Tidsskr Nor Laegeforen. 2013;133(2):165. 23344600 10.4045/tidsskr.12.0494

[22] Nomoto H , Miyoshi H , Nakamura A , et al. A case of osteomalacia due to deranged mineral balance caused by saccharated ferric oxide and short‐bowel syndrome a case report. Medicine. 2017;96(39):1‐4. 10.1097/MD.0000000000008147 PMC5626297 28953654

[23] Poursac N . Hypophosphatémie et FGF23 élevé Hypophosphataemia and elevated FGF23. Rhumatos. 2015;12(105):61‐64.

[24] Reyes M , Diamond T . Hypophosphataemic rickets due to parenteral ferrous carboxymaltose in a young man with Crohn disease and iron deficiency: a case report and review of literature. J Clin Case Rep. 2017;7(02):931.

[25] Gomez Rodriguez S , Castro Ramos JC , Abreu Padin C , Gomez PF . Intravenous iron induced severe hypophophatemia in a gastric bypass patient. Endocrinol Diabetes Nutr (Engl Ed). 2019;66(5):340‐342. 30658902 10.1016/j.endinu.2018.10.006

[26] Sangros Sahun MJ , Goni Girones E , Camarero Salazar A , Estebanez Estebanez C , Lozano Martinez ME . Symptomatic hypophosphataemic osteomalacia secondary to the treatment with iron carboxymaltose detected in bone scintigraphy. Rev Esp Med Nucl Imagen Mol. 2016;35(6):391‐393. 27246291 10.1016/j.remn.2016.04.006

[27] Sato K , Nohtomi K , Demura H , et al. Saccharated ferric oxide (SFO)‐induced osteomalacia: in vitro inhibition by SFO of bone formation and 1,25‐dihydroxy‐vitamin D production in renal tubules. Bone. 1997;21(1):57‐64. 9213008 10.1016/s8756-3282(97)00084-7

[28] Schaefer B , Glodny B , Zoller H . Blood and bone loser. Gastroenterology. 2017;152(6):e5–6. 10.1053/j.gastro.2016.09.050 28384445

[29] Schouten BJ , Doogue MP , Soule SG , Hunt PJ . Iron polymaltose‐induced FGF23 elevation complicated by hypophosphataemic osteomalacia. Ann Clin Biochem. 2009;46(2):167‐169. 19151167 10.1258/acb.2008.008151

[30] Tejera Segura B , Martínez‐Morillo M , Cañellas J , Holgado S . Arthralgias and fractures in an adult male: beyond hypovitaminosis D. Med Clin (Barc). 2014;142(9):423‐424. 24035413 10.1016/j.medcli.2013.06.024

[31] Shimizu Y , Tada Y , Yamauchi M , et al. Hypophosphatemia induced by intravenous administration of saccharated ferric oxide another form of FGF23‐related hypophosphatemia. Bone. 2009;45(4):814‐816. 19555782 10.1016/j.bone.2009.06.017

[32] Tournis S , Michopoulos S , Makris K , Terpos E . Re: hypophosphatemia, severe bone pain, gait disturbance, and fatigue fractures after iron substitution in inflammatory bowel disease: a case report. J Bone Miner Res. 2018;33(3):543‐545. 29281128 10.1002/jbmr.3372

[33] Tozzi D , Tozzi J . Osteomalacia and insufficiency fractures secondary to intravenous iron therapy: a case report. J Orthop Case Rep. 2020;10(1):4‐7. 32547968 10.13107/jocr.2020.v10.i01.1612 PMC7276570

[34] Urbina T , Belkhir R , Rossi G , et al. Iron supplementation‐induced phosphaturic osteomalacia: FGF23 is the culprit. J Bone Miner Res. 2018;33(3):540‐542. 29281120 10.1002/jbmr.3369

[35] Vasquez‐Rios G , Chapel A , Merando A , Philip I , Martin KJ . Life‐threatening hypophosphatemia following intravenous iron infusion. Nefrologia. 2021;41(4):464‐470. 10.1016/j.nefroe.2021.08.006 36165116

[36] Yamamoto S , Okada Y , Mori H , Tanaka Y , Fukumoto S . Fibroblast growth factor 23‐related osteomalacia caused by the prolonged administration of saccharated ferric oxide. Intern Med. 2012;51(17):2375‐2378. 22975552 10.2169/internalmedicine.51.7450

[37] Yamamoto S , Okada Y , Mori H , et al. Iatrogenic osteomalacia: report of two cases. J UOEH. 2013;35(1):25‐31. 23475021 10.7888/juoeh.35.25

[38] Suzuki A , Ohoike H , Matsuoka Y , Irimajiri S . Iatrogenic osteomalacia caused by intravenous administration of saccharated ferric oxide. Am J Hematol. 1993;43(1):75‐76. 8317471 10.1002/ajh.2830430121

[39] Fang W , Rizvi QUA , Garg M , Kenny R , McMahon LP . Hypophosphatemia after ferric carboxymaltose is unrelated to symptoms, intestinal inflammation or vitamin D status. BMC Gastroenterol. 2020;20(1):183. 32522150 10.1186/s12876-020-01298-9 PMC7288415

[40] Schouten BJ , Hunt PJ , Livesey JH , Soule SG , Frampton CM . FGF23 elevation and hypophosphatemia after intravenous iron polymaltose: a prospective study. J Clin Endocrinol Metabol. 2009;94(7):2332‐2337. 10.1210/jc.2008-2396 19366850

[41] Schaefer B , Tobiasch M , Wagner S , et al. Hypophosphatemia after intravenous iron therapy: comprehensive review of clinical findings and recommendations for management. Bone. 2021;154:116202. 34534708 10.1016/j.bone.2021.116202

[42] Wolf F , Poletaev V , Elias M . Does intravenous iron therapy decrease serum phosphorus levels? J Am Soc Nephrol. 2017;28:1105‐1106.

[43] Edmonston D , Wolf M . FGF23 at the crossroads of phosphate, iron economy and erythropoiesis. Nat Rev Nephrol. 2020;16(1):7‐19. 31519999 10.1038/s41581-019-0189-5

[44] Imel EA , Peacock M , Gray AK , Padgett LR , Hui SL , Econs MJ . Iron modifies plasma FGF23 differently in autosomal dominant hypophosphatemic rickets and healthy humans. J Clin Endocrinol Metab. 2011;96(11):3541‐3549. 21880793 10.1210/jc.2011-1239 PMC3205884

[45] Kassianides X , Bhandari S . Hypophosphatemia, fibroblast growth factor 23 and third‐generation intravenous iron compounds: a narrative review. Drugs Context. 2021;10:1‐23. 10.7573/dic.2020-11-3 PMC7819638 33519940

[46] Rosano G , Schiefke I , Goehring U‐M , Fabien V , Bonassi S , Stein J . A pooled analysis of serum phosphate measurements and potential hypophosphatemia events in 45 interventional trials with ferric carboxymaltose. J Clin Med. 2020;9(11):3587. 10.3390/jcm9113587 PMC7694774 33172157

[47] Schaefer B , Tobiasch M , Viveiros A , et al. Hypophosphatemia after treatment of iron deficiency with intravenous ferric carboxymaltose or iron isomaltoside—a systematic review and meta‐analysis. Br J Clin Pharmacol. 2021;87(5):2256‐2273. 33188534 10.1111/bcp.14643 PMC8247006

[48] Glaspy JA , Lim‐Watson MZ , Libre MA , et al. Hypophosphatemia associated with intravenous iron therapies for iron deficiency anemia: a systematic literature review. Ther Clin Risk Manag. 2020;16:245‐259. 32308402 10.2147/TCRM.S243462 PMC7152545

[49] MHRA . Ferric carboxymaltose (Ferrinject) risk of symptomatic hypophosphatemia leading to osteomalacia and fractures [Internet]. 2020. Available at: https://www.gov.uk/drug-safety-update/ferric-carboxymaltose-ferinject-risk-of-symptomatic-hypophosphatemia-leading-to-osteomalacia-and-fractures.

[50] Rosen CJ , Gallagher JC . The 2011 IOM report on vitamin D and calcium requirements for North America: clinical implications for providers treating patients with low bone mineral density. J Clin Densitom. 2011;14(2):79‐84. 21787514 10.1016/j.jocd.2011.03.004

[51] Patterson CR. Metabolic disorders of bone. Oxford: Blackwell Scientific Publications; 1974.

[52] Bager P , Hvas CL , Dahlerup JF . Drug‐specific hypophosphatemia and hypersensitivity reactions following different intravenous iron infusions. Br J Clin Pharmacol. 2017;83(5):1118‐1125. 27859495 10.1111/bcp.13189 PMC5401972

