# Supplementary material for: Osteomalacia as a Complication of Intravenous Iron Infusion: A Systematic Review of Case Reports
Source: J Bone Miner Res. 2022 May 7;37(6):1188–99. doi: 10.1002/jbmr.4558 (PMC9322686; doi:10.1002/jbmr.4558)

Appendix 1

Iron review search strategy

Database: Ovid MEDLINE(R) and Epub Ahead of Print, In-Process, In-Data-Review & Other Non-Indexed Citations and Daily <1946 to March 19, 2021>

Search Strategy:

--------------------------------------------------------------------------------

1 Iron/ (96553)

2 infusion.mp. (233284)

3 Infusions, Intravenous/ or Administration, Intravenous/ or intravenous.mp. or Injections, Intravenous/ (401936)

4 2 or 3 (563237)

5 1 and 4 (3042)

6 Humans/ or exp Hypophosphatemia/ or Osteomalacia/ or hypophosphat*mia.mp. (19111482)

7 Phosphates/bl, df [Blood, Deficiency] (9000)

8 osteomalacia.mp. or exp Osteomalacia/ (7129)

9 6 or 7 or 8 (19114159)

10 Bone Remodeling/ or "Bone and Bones"/ or Female/ or Male/ or exp Biomarkers/ or Humans/ or bone turnover marker.mp. or Osteoporosis/ (21322167)

11 alkaline phosphatase.mp. or exp Alkaline Phosphatase/ (94516)

12 Vitamin D/ or vitamin D.mp. (75850)

13 fracture.mp. (216219)

14 "Bone and Bones"/ or Osteomalacia/ or Humans/ or pseudo fracture.mp. or Fractures, Bone/ (19144551)

15 fracture healing.mp. or Fracture Healing/ (18191)

16 Fractures, Ununited/ or fracture nonunion.mp. (6345)

17 bone mineral density.mp. or Bone Density/ (67555)

18 X ray.mp. or X-Rays/ (837572)

19 10 or 11 or 12 or 13 or 14 or 15 or 16 or 17 or 18 (21694638)

20 case report.mp. or Case Reports/ (2223627)

21 5 and 9 (2297)

22 5 and 19 (2771)

23 20 and 21 (125)

24 21 or 22 or 23 (2771)

Search strategy Web of science

| Search History | | | | |
| --- | --- | --- | --- | --- |
| **Set** | **Results** | **Save History / Create AlertOpen Saved History** | **Combine Sets**  **AND  OR**  **Combine** | **Delete Sets**  **Select All  Delete** |
|  | | | | |
| # 23 | [**721**](http://apps.webofknowledge.com/summary.do?product=UA&doc=1&qid=32&SID=D2YGASj8w6yH5oo1GEB&search_mode=CombineSearches&update_back2search_link_param=yes) | #22 OR #21 OR #20  *Databases= WOS, BCI, BIOSIS, CCC, DRCI, DIIDW, KJD, MEDLINE, RSCI, SCIELO, ZOOREC Timespan=All years*  *Search language=Auto* |  |  |
|  | | | | |
| # 22 | [**30**](http://apps.webofknowledge.com/summary.do?product=UA&doc=1&qid=31&SID=D2YGASj8w6yH5oo1GEB&search_mode=AdvancedSearch&update_back2search_link_param=yes) | #19 AND #20  *Databases= WOS, BCI, BIOSIS, CCC, DRCI, DIIDW, KJD, MEDLINE, RSCI, SCIELO, ZOOREC Timespan=All years*  *Search language=Auto* |  |  |
|  | | | | |
| # 21 | [**602**](http://apps.webofknowledge.com/summary.do?product=UA&doc=1&qid=30&SID=D2YGASj8w6yH5oo1GEB&search_mode=AdvancedSearch&update_back2search_link_param=yes) | #5 AND#18  *Databases= WOS, BCI, BIOSIS, CCC, DRCI, DIIDW, KJD, MEDLINE, RSCI, SCIELO, ZOOREC Timespan=All years*  *Search language=Auto* |  |  |
|  | | | | |
| # 20 | [**167**](http://apps.webofknowledge.com/summary.do?product=UA&doc=1&qid=27&SID=D2YGASj8w6yH5oo1GEB&search_mode=AdvancedSearch&update_back2search_link_param=yes) | #5 AND #9  *Databases= WOS, BCI, BIOSIS, CCC, DRCI, DIIDW, KJD, MEDLINE, RSCI, SCIELO, ZOOREC Timespan=All years*  *Search language=Auto* |  |  |
|  | | | | |
| # 19 | [**1,904,587**](http://apps.webofknowledge.com/summary.do?product=UA&doc=1&qid=26&SID=D2YGASj8w6yH5oo1GEB&search_mode=AdvancedSearch&update_back2search_link_param=yes) | TS=case report  *Databases= WOS, BCI, BIOSIS, CCC, DRCI, DIIDW, KJD, MEDLINE, RSCI, SCIELO, ZOOREC Timespan=All years*  *Search language=Auto* |  |  |
|  | | | | |
| # 18 | [**4,026,847**](http://apps.webofknowledge.com/summary.do?product=UA&doc=1&qid=25&SID=D2YGASj8w6yH5oo1GEB&search_mode=CombineSearches&update_back2search_link_param=yes) | #17 OR #16 OR #15 OR #14 OR #13 OR #12 OR #11 OR #10  *Databases= WOS, BCI, BIOSIS, CCC, DRCI, DIIDW, KJD, MEDLINE, RSCI, SCIELO, ZOOREC Timespan=All years*  *Search language=Auto* |  |  |
|  | | | | |
| # 17 | [**2,671,398**](http://apps.webofknowledge.com/summary.do?product=UA&doc=1&qid=24&SID=D2YGASj8w6yH5oo1GEB&search_mode=AdvancedSearch&update_back2search_link_param=yes) | TS= X-ray  *Databases= WOS, BCI, BIOSIS, CCC, DRCI, DIIDW, KJD, MEDLINE, RSCI, SCIELO, ZOOREC Timespan=All years*  *Search language=Auto* |  |  |
|  | | | | |
| # 16 | [**3,190**](http://apps.webofknowledge.com/summary.do?product=UA&doc=1&qid=23&SID=D2YGASj8w6yH5oo1GEB&search_mode=AdvancedSearch&update_back2search_link_param=yes) | TS= pseudo fracture  *Databases= WOS, BCI, BIOSIS, CCC, DRCI, DIIDW, KJD, MEDLINE, RSCI, SCIELO, ZOOREC Timespan=All years*  *Search language=Auto* |  |  |
|  | | | | |
| # 15 | [**3,718**](http://apps.webofknowledge.com/summary.do?product=UA&doc=1&qid=18&SID=D2YGASj8w6yH5oo1GEB&search_mode=AdvancedSearch&update_back2search_link_param=yes) | TS= malunion fracture  *Databases= WOS, BCI, BIOSIS, CCC, DRCI, DIIDW, KJD, MEDLINE, RSCI, SCIELO, ZOOREC Timespan=All years*  *Search language=Auto* |  |  |
|  | | | | |
| # 14 | [**10,597**](http://apps.webofknowledge.com/summary.do?product=UA&doc=1&qid=17&SID=D2YGASj8w6yH5oo1GEB&search_mode=AdvancedSearch&update_back2search_link_param=yes) | TS=nonunion fracture  *Databases= WOS, BCI, BIOSIS, CCC, DRCI, DIIDW, KJD, MEDLINE, RSCI, SCIELO, ZOOREC Timespan=All years*  *Search language=Auto* |  |  |
|  | | | | |
| # 13 | [**1,101,308**](http://apps.webofknowledge.com/summary.do?product=UA&doc=1&qid=16&SID=D2YGASj8w6yH5oo1GEB&search_mode=AdvancedSearch&update_back2search_link_param=yes) | TS= fracture  *Databases= WOS, BCI, BIOSIS, CCC, DRCI, DIIDW, KJD, MEDLINE, RSCI, SCIELO, ZOOREC Timespan=All years*  *Search language=Auto* |  |  |
|  | | | | |
| # 12 | [**220,514**](http://apps.webofknowledge.com/summary.do?product=UA&doc=1&qid=15&SID=D2YGASj8w6yH5oo1GEB&search_mode=AdvancedSearch&update_back2search_link_param=yes) | TS= vitamin D  *Databases= WOS, BCI, BIOSIS, CCC, DRCI, DIIDW, KJD, MEDLINE, RSCI, SCIELO, ZOOREC Timespan=All years*  *Search language=Auto* |  |  |
|  | | | | |
| # 11 | [**164,445**](http://apps.webofknowledge.com/summary.do?product=UA&doc=1&qid=14&SID=D2YGASj8w6yH5oo1GEB&search_mode=AdvancedSearch&update_back2search_link_param=yes) | TS= alkaline phosphatase  *Databases= WOS, BCI, BIOSIS, CCC, DRCI, DIIDW, KJD, MEDLINE, RSCI, SCIELO, ZOOREC Timespan=All years*  *Search language=Auto* |  |  |
|  | | | | |
| # 10 | [**13,766**](http://apps.webofknowledge.com/summary.do?product=UA&doc=1&qid=13&SID=D2YGASj8w6yH5oo1GEB&search_mode=AdvancedSearch&update_back2search_link_param=yes) | TS= bone turnover marker  *Databases= WOS, BCI, BIOSIS, CCC, DRCI, DIIDW, KJD, MEDLINE, RSCI, SCIELO, ZOOREC Timespan=All years*  *Search language=Auto* |  |  |
|  | | | | |
| # 9 | [**22,339**](http://apps.webofknowledge.com/summary.do?product=UA&doc=1&qid=12&SID=D2YGASj8w6yH5oo1GEB&search_mode=CombineSearches&update_back2search_link_param=yes) | #8 OR #7 OR #6  *Databases= WOS, BCI, BIOSIS, CCC, DRCI, DIIDW, KJD, MEDLINE, RSCI, SCIELO, ZOOREC Timespan=All years*  *Search language=Auto* |  |  |
|  | | | | |
| # 8 | [**12,405**](http://apps.webofknowledge.com/summary.do?product=UA&doc=1&qid=11&SID=D2YGASj8w6yH5oo1GEB&search_mode=AdvancedSearch&update_back2search_link_param=yes) | TS= osteomalacia  *Databases= WOS, BCI, BIOSIS, CCC, DRCI, DIIDW, KJD, MEDLINE, RSCI, SCIELO, ZOOREC Timespan=All years*  *Search language=Auto* |  |  |
|  | | | | |
| # 7 | [**2,258**](http://apps.webofknowledge.com/summary.do?product=UA&doc=1&qid=10&SID=D2YGASj8w6yH5oo1GEB&search_mode=AdvancedSearch&update_back2search_link_param=yes) | TS= "low phosphate"  *Databases= WOS, BCI, BIOSIS, CCC, DRCI, DIIDW, KJD, MEDLINE, RSCI, SCIELO, ZOOREC Timespan=All years*  *Search language=Auto* |  |  |
|  | | | | |
| # 6 | [**9,613**](http://apps.webofknowledge.com/summary.do?product=UA&doc=1&qid=9&SID=D2YGASj8w6yH5oo1GEB&search_mode=AdvancedSearch&update_back2search_link_param=yes) | TS= Hypophosphat*mia  *Databases= WOS, BCI, BIOSIS, CCC, DRCI, DIIDW, KJD, MEDLINE, RSCI, SCIELO, ZOOREC Timespan=All years*  *Search language=Auto* |  |  |
|  | | | | |
| # 5 | [**13,419**](http://apps.webofknowledge.com/summary.do?product=UA&doc=1&qid=7&SID=D2YGASj8w6yH5oo1GEB&search_mode=CombineSearches&update_back2search_link_param=yes) | #4 AND #1  *Databases= WOS, BCI, BIOSIS, CCC, DRCI, DIIDW, KJD, MEDLINE, RSCI, SCIELO, ZOOREC Timespan=All years*  *Search language=Auto* |  |  |
|  | | | | |
| # 4 | [**990,149**](http://apps.webofknowledge.com/summary.do?product=UA&doc=1&qid=4&SID=D2YGASj8w6yH5oo1GEB&search_mode=CombineSearches&update_back2search_link_param=yes) | #3 OR #2  *Databases= WOS, BCI, BIOSIS, CCC, DRCI, DIIDW, KJD, MEDLINE, RSCI, SCIELO, ZOOREC Timespan=All years*  *Search language=Auto* |  |  |
|  | | | | |
| # 3 | [**604,914**](http://apps.webofknowledge.com/summary.do?product=UA&doc=1&qid=3&SID=D2YGASj8w6yH5oo1GEB&search_mode=AdvancedSearch&update_back2search_link_param=yes) | TS=intravenous  *Databases= WOS, BCI, BIOSIS, CCC, DRCI, DIIDW, KJD, MEDLINE, RSCI, SCIELO, ZOOREC Timespan=All years*  *Search language=Auto* |  |  |
|  | | | | |
| # 2 | [**522,475**](http://apps.webofknowledge.com/summary.do?product=UA&doc=1&qid=2&SID=D2YGASj8w6yH5oo1GEB&search_mode=AdvancedSearch&update_back2search_link_param=yes) | TS=infusion  *Databases= WOS, BCI, BIOSIS, CCC, DRCI, DIIDW, KJD, MEDLINE, RSCI, SCIELO, ZOOREC Timespan=All years*  *Search language=Auto* |  |  |
|  | | | | |
| # 1 | [**1,918,600**](http://apps.webofknowledge.com/summary.do?product=UA&doc=1&qid=1&SID=D2YGASj8w6yH5oo1GEB&search_mode=AdvancedSearch&update_back2search_link_param=yes) | TS= iron  *Databases= WOS, BCI, BIOSIS, CCC, DRCI, DIIDW, KJD, MEDLINE, RSCI, SCIELO, ZOOREC Timespan=All years*  *Search language=Auto* |  |  |

Search strategy Embase

Strategy 998582

| **#** | **Database** | **Search term** | **Results** |
| --- | --- | --- | --- |
| 1 | EMBASE | (iron).ti,ab | 228715 |
| 2 | EMBASE | (infusion).ti,ab | 313890 |
| 4 | EMBASE | (intravenous).ti,ab | 389440 |
| 5 | EMBASE | (2 OR 4) | 634691 |
| 6 | EMBASE | (1 AND 5) | 8665 |
| 8 | EMBASE | exp HYPOPHOSPHATEMIA/ OR "HYPOPHOSPHATEMIC OSTEOMALACIA"/ | 11993 |
| 9 | EMBASE | exp OSTEOMALACIA/ | 9277 |
| 10 | EMBASE | "PHOSPHATE BLOOD LEVEL"/ OR exp "PHOSPHATE DEFICIENCY"/ | 17408 |
| 11 | EMBASE | (calcium).ti,ab | 467953 |
| 12 | EMBASE | (bone turnover marker).ti,ab | 464 |
| 13 | EMBASE | (vitamin D).ti,ab | 96691 |
| 14 | EMBASE | (fracture).ti,ab | 216759 |
| 15 | EMBASE | (pseudofracture).ti,ab | 95 |
| 16 | EMBASE | "FRACTURE HEALING"/ OR "FRACTURE NONUNION"/ OR "FRACTURE HEALING IMPAIRMENT"/ OR "FRACTURE MALUNION"/ | 38731 |
| 17 | EMBASE | "BONE DENSITY"/ OR "BONE CHARACTERISTICS AND FUNCTIONS"/ | 98852 |
| 18 | EMBASE | "X RAY"/ | 71896 |
| 19 | EMBASE | (11 OR 12 OR 13 OR 14 OR 15 OR 16 OR 17 OR 18) | 877305 |
| 20 | EMBASE | "CASE REPORT"/ OR "CASE SERIES"/ OR "CASE STUDIES"/ OR "CASE STUDY"/ | 2664804 |
| 21 | EMBASE | (8 OR 9) | 19162 |
| 23 | EMBASE | (6 AND 21) | 193 |
| 25 | EMBASE | "ALKALINE PHOSPHATASE"/ | 110961 |
| 26 | EMBASE | (19 OR 25) | 968586 |
| 27 | EMBASE | (6 AND 26) | 416 |
| 29 | EMBASE | (20 AND 23) | 45 |
| 31 | EMBASE | (8 OR 9 OR 10) | 34280 |
| 32 | EMBASE | (6 AND 31) | 263 |
| 33 | EMBASE | (27 OR 29 OR 32) | 607 |

Cochrane search strategy


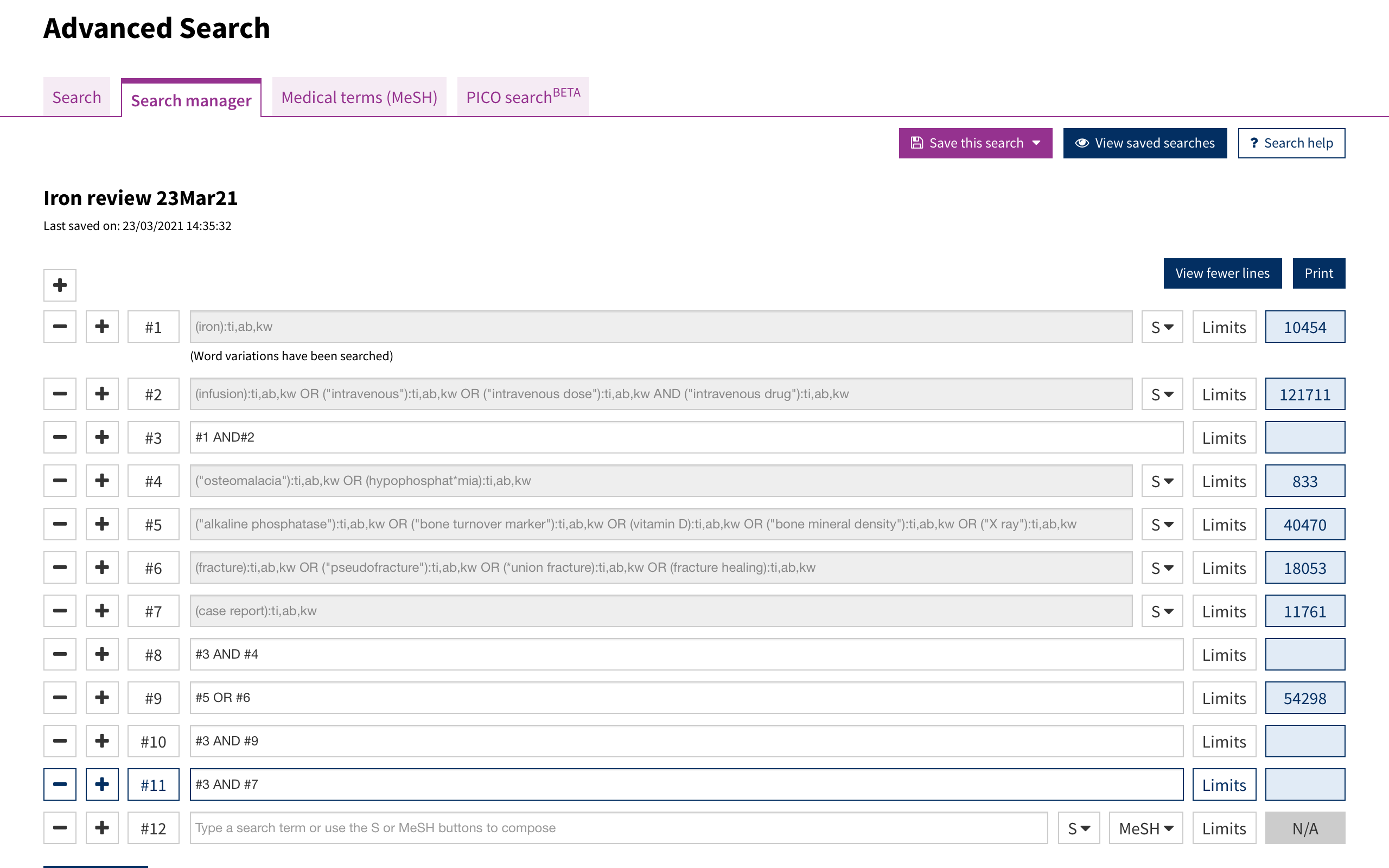

Supplement: Supplementary file 1 — Appendix S1: Supporting Information [file JBMR-37-1188-s003.docx]
