# Supplementary material for: Osteomalacia as a Complication of Intravenous Iron Infusion: A Systematic Review of Case Reports
Source: J Bone Miner Res. 2022 May 7;37(6):1188–99. doi: 10.1002/jbmr.4558 (PMC9322686; doi:10.1002/jbmr.4558)
Supplement: Supplementary file 2 — Appendix S2: Supporting Information [file JBMR-37-1188-s002.docx]

Data extraction form

| Study number |
| --- |
| First author |
| Year |
| Country |
| Title |
| Reference |
| Study design |
| Method of enrolment  1) isolated case report 2) multiple cases  3) cohort  4) registry review  5) other |
| Cause of iron deficiency  1) CKD 2) Inflammatory Bowel Disease 3)Gynaecological bleeding 4) Pregnancy 5) other- specify |
| Age (y) |
| Gender |
| key clinical features of the condition that led to iron deficiency |
| Previous health history (comorbidities) |
| Previous skeletal condition (TV Jun) |
| Is the patient affected by a condition that affects the skeleton? Y/N |
| If yes, with condition  1) CKD 2) malabsorption 3)glucocorticoid use 4) Vitamin D Deficiency  5) others- specify |
| which Intravenous iron therapy |
| Iron dose (per infusion) |
| number of infusions |
| If several infusions, interval between infusions |
| Cumulative iron dose |
| Other medications in use |
| Presentation symptom |
| Complaints/ symptoms/ reason for referral associated with iron infusion |
| Lowest P level |
| Phosphate unit |
| reference range |
| Phosphate in mmol/L |
| Phosphate level in mg/L |
| HipoP duration |
| HipoP management |
| intact FGF23 normal/ abnormal/ nonreported |
| FGF23 value |
| FGF23 unit |
| FGF23 reference range |
| iFGF23 in times the upper reference range |
| c-terminal FGF23 |
| 25OH vitamin D normal/ abnormal/ nonreported |
| 25OHD vaule |
| 25OHD unit |
| 25OHD reference range |
| 1,25(OH)2D normal, abnormal, nonreported |
| 1,25(OH)2D value |
| 1,25(OH)2D unit |
| 1,25(OH)2D reference range |
| PTH normal/ abnormal/ nonreported |
| PTH value |
| PTH unit |
| PTH reference range |
| Fractional P urinary excretion |
| Calcium  normal/ abnormal/ nonreported |
| Calcium value |
| Calcium unit |
| calcium reference range |
| Which Calcium (total or album corrected)? |
| ALP normal/ abnormal/ nonreported |
| ALP value |
| ALP unit |
| ALP reference range |
| Liver function |
| Bone pain (Y/N) |
| Pain not associated with fracture vs pain associated with fracture |
| Pain site |
| Pain localization |
| Bone pain, if yes characterise |
| Fractures (Y/N) |
| Fracture site |
| Fractures, if yes characterise |
| Pseudofractures (Y/N) |
| Pseudofracture site |
| Pseudofractures if Y characterise |
| Muscular signs or symptoms (Y/N) |
| Muscular signs/ Symptoms if Y characterise |
| Other symptoms |
| Bone turnover markers (other than ALP) |
| DXA category |
| DXA (results and temporal relationship with iron infusion/ hypoP |
| Isotope bone scan (yes/no) |
| Isotope bone scans (result) |
| Bone biopsy |
| Other tests or scans |
| HypoP history details |
| HypoP treatment (Y/N) |
| Hypo Treatment |
| Iron treatment management |
| Time to hypoP resolution |
| Time to symptoms resolution |
| Details of outcome |
| Patient outcome |
| Other comments |
| HypoP management |
| Reviewer check |
