# Supplementary material for: Osteomalacia as a Complication of Intravenous Iron Infusion: A Systematic Review of Case Reports
Source: J Bone Miner Res. 2022 May 7;37(6):1188–99. doi: 10.1002/jbmr.4558 (PMC9322686; doi:10.1002/jbmr.4558)
Supplement: Supplementary file 3 — Appendix S3: Supporting Information [file JBMR-37-1188-s001.docx]

Quality assessment form

| Study number |
| --- |
| First author |
| Year |
| Title |
| 1. Does the patient(s) represent(s) the whole experience of the investigator (centre) or is the selection method unclear to the extent that other patients with similar presentation may not have been reported? Whole experience * Selection method unclear |
| Justification |
| 2. Was the exposure adequately ascertained?  clinical records * administrative/billing codes self-report  not reported |
| Justification |
| 3. Was the outcome adequately ascertained?   clinical records* administrative/ billing codes* self-report not reported |
| Justification |
| 4. Were other alternative causes that may explain the observation ruled out? Yes* No |
| Justification |
| 5. Was there a challenge/rechallenge phenomenon? Yes* No |
| Justification |
| 6. Was there a dose–response effect? Yes* No Non-reported |
| Justification |
| 7. Was follow-up long enough for outcomes to occur? Yes* No |
| Justification |
| 8. Is the case(s) described with sufficient details to allow other investigators to replicate the research or to allow practitioners make inferences related to their own practice? Yes* No |
| Justification |
